# Water Uptake in Epoxy Ionic Liquid Free Film Polymer by Gravimetric Analysis and Comparison with Nondestructive Dielectric Analysis

**DOI:** 10.3390/nano12040651

**Published:** 2022-02-15

**Authors:** Lucas Ollivier-Lamarque, Sébastien Livi, Tetsuya Uchimoto, Nicolas Mary

**Affiliations:** 1 ELyTMaX IRL3757, CNRS, Université de Lyon, INSA Lyon, Centrale Lyon, Université Claude Bernard Lyon 1, Tohoku University, Sendai 980-8577, Japan; lucas.ollivier-lamarque@insa-lyon.fr; 2 Institute of Fluid Science, Tohoku University, Sendai 980-8577, Japan; 3 Graduate School of Engineering, Tohoku University, Sendai 980-8577, Japan; 4 Université de Lyon, CNRS, UMR 5223, Ingénierie des Matériaux Polymères, Université Claude Bernard Lyon 1, INSA Lyon, Université Jean Monnet, CEDEX, F-69621 Villeurbanne, France; sebastien.livi@insa-lyon.fr; 5 Université de Lyon, INSA Lyon, Université Claude Bernard Lyon 1, CNRS, MATEIS, UMR5510, F-69621 Villeurbanne, France

**Keywords:** epoxy prepolymers, ionic liquid, dielectric measurements, gravimetric measurements, water uptake

## Abstract

Due to their high surface coverage, good adhesion to metal surfaces, and their excellent corrosion resistance, epoxy thermosets are widely used as protective coatings. However, anticorrosion protection of these coatings can be improved against water uptake and can be tuned by changing the chemical nature of the curing agents. In this work, a comparative study has been performed on the water uptake of an epoxy–amine based on bisphenol A diglycidyl ether (DGEBA) cured with an aliphatic amine and the same epoxy initiated with a phosphonium ionic liquid (IL). Thus, the epoxy networks were immersed in saline water solution in a controlled temperature environment. Gravimetric and electric impedance measurements were carried out for a maximum of 3 months. Results were analyzed in order to assess the water diffusion coefficients and water saturation limits. Two models, the Brasher–Kingsbury and a novel mixing rule, were applied on permittivity values. Results highlighted that epoxy–ionic liquid systems are less sensitive to water uptake than conventional epoxy–amine networks. Due to their higher hydrophobic properties the water diffusion coefficient of epoxy–ionic liquid systems are two times less compared to epoxy–amine samples and the water saturation limit is more than four times less. The analysis also shows that the novel mixing rule model proposed here is prone to better estimate the water uptake with accuracy from electrical impedance measurements.

## 1. Introduction

Corrosion reduces structures’ and components’ life span in the transportation, energy, utilities, and civil engineering domains. Several strategies, such as structural transformations and surface treatments, can be applied to prevent degradation. Metallic or organic coatings are other available solutions. These latter act as physical barriers between the substrate and the environment. Organic coatings present good strength-to-weight ratio; they are easy to apply and cost-efficient [1,2,3]. Nowadays, epoxy prepolymers are widely used for anticorrosion protection due to their high surface coverage, good adhesion to metal surfaces and chemical properties, especially their excellent corrosion resistance. However, the protective performance of epoxy resin coating can be improved. In fact, microcracks, and holes can be generated during the curing process of the epoxy coatings due to their high crosslinking density [3,4]. In addition, the pinhole porosity of epoxy coatings is well-known to create a perfect way for the diffusion of corrosive medium molecules, such as water, oxygen, and ions, into the networks reducing their anticorrosion performances [4]. For these reasons, various strategies have been reported to delay the penetration of corrosive medium [3,4]: (i) to create the tortuosity of corrosion medium diffusion path and reduces the porosity of epoxy resin coating; (ii) to reduce the affinity between corrosive medium and epoxy resin coating; and (iii) to inhibit the reaction between corrosive medium and metal surface. To take up these challenges, ionic liquids (ILs) offer real opportunities to design new (multi)functional-dedicated epoxy surface coatings with enhanced properties such as thermal stability, mechanical performances, and water vapor barrier properties. Moreover, phosphonium ILs can be used as new alternatives to the conventional amine or anhydride hardeners for crosslinking epoxy networks. Various research groups have demonstrated that ILs combined with basic counter anions such as dicyanamide, phosphinate, or phosphate anions could initiate the epoxy polymerization. They offer the opportunity to design reactive systems having a tunable reactivity (from conventional polymerization times to ‘fast-cure’ systems [5,6,7]. Such a route leads to new epoxy–IL networks displaying good mechanical performances, including high thermal stability under nitrogen (>400–450 °C), a hydrophobic surface behavior, and a T_g_ included between 80 and 170 °C, which can be tuned from the chemical nature of the anions [8,9]. Even though they have excellent barrier properties, water diffusion still occurs, and hence corrosion may appear on the metallic substrate [10,11]. When water diffuses in the polymer layer, it first introduces carbonyl groups on the epoxy chain. Then, hydrolyses on the carbonyl group lead to chain scission and water barrier properties dropping [12,13]. Therefore, it is essential to quantify the water uptake for improvement of coatings’ performances.

Gravimetric measurements (mass difference between dry and wet samples as function of immersion time) are associated with data treatment using Fick’s diffusion law to calculate the saturation and coefficient of water diffusion in the polymer [14]. Fick’s diffusion law assumes a flux of species establishes from the high concentration region to low concentration with a concentration gradient. For incompressible media as polymer materials, Fick’s second law is preferred to access the concentration profile of diffusible species as a function of time [15,16,17]. Equation (1) reports a general solution of the diffusion and the saturation phenomena valid for water [18]. At first, samples are supposed dehydrated at the initial state. When immersed, the sample surfaces adsorb water molecules with time ($\chi_{t}$) until saturation ($\chi_{sat}$). Therefore, a water gradient is established between the surface to the volume of polymer. Based on these assumptions, Crank et al. proposed a partial differential equation solution for thin samples for which edge effects are neglected because of the infinite surfaces hypothesis (Equation (1)). According to Kim et al., Equation (1) can be simplified to Equation (2) for an easier calculation implementation without generating large errors. Note that the water uptake can be considered proportional to the square root of time for short time immersion tests [18]. In this paper, for a better visualization, the choice has been made to plot the diffusion curves as a function of square root of time.

(1) $$\frac{\chi_{t}}{\chi_{sat}}=1-\frac{8}{\pi^{2}}\sum_{n=0}^{\infty }\frac{1}{{\left(2n+1\right)}^{2}}\exp\left(\frac{-D_{w}t{\left(2n+1\right)}^{2}\pi^{2}}{\delta^{2}}\right),$$

(2) $$\frac{\chi_{t}}{\chi_{sat}}=1-\exp\left(-7.3{\left(\frac{D_{w}t}{\delta^{2}}\right)}^{0.75}\right),$$

where $D_{w}$ is the coefficient of diffusion (cm^2^/s) of water, t is the immersion time, and δ is the sample thickness (cm).

Water uptake influences the material’s permittivity (*ε_t_*) which is an intrinsic dielectric property depending on the material’s microstructure, chemical composition, etc. [19,20]. It expresses the material’s ability to organize electric dipoles under an applied electric field and changes obviously with water molecules in the polymer volume, for instance. In the literature, the water permittivity (*ε_w_*) is about 80 (as in Brasher–Kingsbury law) whereas it is about 2 to 4 (*ε_epoxy_*) for epoxy materials [21]. Whatever the sample shape, the permittivity is proportional to capacitance (*C*). This latter can be determined from electrical or electrochemical impedance tests [22]. For parallel-plate geometry, the capacitance–permittivity relation equation is *C = ε·S/d* where *S* is the smallest electrode surface and d is the distance between the two electrodes (i.e., the polymer thickness sample in this work). To neglect mass transfer phenomena regarding charge transfer, experiments are often carried out at a single frequency analysis of 10 kHz for capacitance (*C*_10Hz_) determinations by electrochemical impedances spectroscopy (EIS). Alternatively, EIS from 100 MHz to 1 Hz are used to determined effective capacitances (*C_eff_*). *C_eff_* is a nondependent frequency capacitance value derived from impedance equivalent circuit on the tested frequency range. For this method, impedance diagrams are usually fitted with an electrical equivalent circuit using constant phase elements (CPE); CPE adjusts variations from ideal capacitance *C_eff_*. Relations between *C_eff_* and CPE parameters are given by Brug [23] or Hsu-Mansfeld [24]. The Brug model assumes a capacitance distribution due to volume inhomogeneities whereas the Hsu-Mansfeld model supposes a normal time constant distribution of resistivity and/or dielectric constant. Additionally, the power-law model supposes a nonuniform distribution of the impedance related to a resistivity profile [25,26]. When the capacitance (*C_10Hz_* or *C_eff_*) is known, the relative permittivity can be calculated and the water uptake is estimated with the Brasher and Kingsbury (B-K) equation, Equation (3)) [27].

(3) χV=100log(εtε0)log(εw),

In Equation (3), $\chi_{V}$, $\varepsilon_{t}$, $\varepsilon_{0}$, and $\varepsilon_{w}$ are the volume water uptake, the relative permittivity at immersion time *t*, the relative permittivity at initial condition (dry condition), and the relative water permittivity at a given frequency, respectively.

B-K equation assumes that (i) the overall capacitance is an average of the local volume capacitance, (ii) the solution does not modify the capacitance and its changes depend on the water penetration only, (iii) the absorbed water permittivity remains at 80, (iv) the water distribution in volume is uniform, (v) the film is free of polar solvent (vi) no swelling occurs [18]. Because of these hypotheses, water uptake deviations are often reported from other approaches [28,29]. Vosgien et al. confirmed the water uptake’s overestimation with the Brasher and Kingsbury’s equation from EIS analysis when swelling is not considered [21]. Therefore, this approach is suitable for comparison between several sets of materials immersed in similar conditions [22]. 

Instead of *C_eff_* and B-K model, permittivity and mixing rules can be used to calculate the water uptake [16]. Mixing rules describe the spatially-distributed permittivity across the material using the frequency dependence with the Cole–Cole relaxation given in Equation (4). It is worth noticing that the mixing rule is tuned according the material microstructure, composition [30,31,32]

(4) $$\varepsilon \left(\omega \right)=\mathbb{R}\left[\varepsilon_{0}+\frac{\varepsilon_{0}-\varepsilon_{\infty }}{1+{\left(j\frac{\omega }{\omega_{c}}\right)}^{1-\alpha }}\right],$$

In Equation (4), ℝ represents the real part of the expression, $\varepsilon_{0}$ and $\varepsilon_{\infty }$ are respectively the permittivity at low frequency and high frequency, ω, $\omega_{c}$, and α are the angular frequency (2πf), the cut-off angular frequency and the Cole–Cole coefficient, respectively [33,34].

For an epoxy amine polymer disc, Ollivier-Lamarque et al. selected the mixing rule reported in Equation (5), where an equivalent permittivity of a two phases material at local scale is supposed [18]. In Equation (5), $\varepsilon_{water}\left(\omega \right)$ and $\varepsilon_{polymer}\left(\omega \right)$ are the permittivity of the primary phase (polymer material) and secondary phase (water), respectively. ε(x,ω,t) becomes the local equivalent permittivity at the position x along with the thickness and immersion time *t*. In Equation (5), δ(x,t) is space and time-dependent volume phase ratio distribution, derived from Fick’s law.

(5) $$\varepsilon \left(x,\omega ,t\right)=\delta \left(x,t\right)\varepsilon_{polymer}\left(\omega \right)+\left[1-\delta \left(x,t\right)\right]\varepsilon_{water}\left(\omega \right),$$

To move from the permittivity to the volume percentage of water, the water diffusion coefficient in the material has to be known. In absence of data in the literature, δ(x,t) is easily computed from experimental data of gravimetric analysis and derivate from the Crank solution of Fick’s second law [14]. A conversion process is given in Equation (6) to turn the value from mass percentage to volume percentage of water.

(6) $$\frac{1}{\delta \left(x,t=\infty \right)}=1+\frac{\rho_{wat}}{\rho_{coat}}\frac{1}{\chi_{m}},$$

In Equation (6), $\chi_{m}$ is the maximum value of the mass percentage of water uptake, $\rho_{wat}$ and $\rho_{coat}$ are the density of water and polymer, respectively

Among the above solutions to quantify the water uptake of polymer materials, gravimetric method is a direct measurement that overrides material considerations (microstructure, composition). However, for on-site monitoring it requires coupons that are not always realistic or do not fit with the installation. Therefore, water uptake estimation by impedance measurements is considered. Because of the B-K model’s limitation, mixing rule approaches combined with permittivity measurements are then attractive solutions for larger material comparisons. 

Nowadays, diamine groups are usually used as hardener for epoxy–coating materials, but they can be replaced by ionic liquids, namely IL [6,35,36,37]. Compared with epoxy–amine systems, epoxy–IL networks show higher hydrophobic properties with contact angle measurements suggesting different water uptake initiation and propagation mechanisms. It is also reported that the contact angle differed from the IL percentage in epoxy, suggesting an effect of the hardener concentration in the water barrier properties.

The present paper aims to evaluate for the first time the opportunity to substitute epoxy–amine systems by epoxy–IL networks for water barrier properties issues and also to discuss results obtained from gravimetric and dielectric measurements. Experiments were performed on freestanding film to overcome the metal substrate and polymer layer interface issues. The gravimetric results were fitted with the second Fick’s law to assess the water diffusion coefficient and saturation value for each film. Dielectric measurements were treated either in term of mixing rules or B-K equations.

## 2. Materials and Methods

### 2.1. Materials

Epoxy–amine and epoxy–ionic liquid samples were prepared with the diglycidyl ether of bisphenol A (DGEBA) based epoxy prepolymer (Epon 828, Hexion Co, Louvain-la-Neuve, Belgium). A conventional aliphatic diamine denoted Jeffamine D400 (AHEW = 400 g·mol^−1^, Huntsman, The Woodlands, TX, USA) was used as curing agent to prepare the epoxy–amine system considered as reference in this paper. In addition, a phosphonium ionic liquid supplied by Solvay denoted IL105 (trihexyl(tetradecyl)-phosphonium 2-ethylhexanoate) was used as initiator of epoxy prepolymer to prepare epoxy–ionic liquid network. 

### 2.2. Specimen Preparation

The epoxy–amine samples were prepared at a stoichiometric ratio of 63 phr of diamine in order to obtain a fully cured network. The curing heat treatment consisted of a temperature ramp of 2 h at 80 °C, 3 h at 120 °C, then 2 h of cooling at room temperature. The epoxy–IL samples were prepared considering a mixing ratio of 10 phr (IL-10) without exudation phenomenon. A specific curing heat treatment in oven was applied for IL samples: 2 h at 80 °C and 3 h at 140 °C, then 2 h of cooling at room temperature. Previous studies investigated the determination of the conversion of epoxide groups in both epoxy IL and epoxy diamine networks. For the epoxy–IL105; is the epoxy conversion was around 94 ± 1% [5] and for the epoxy–D400 around 96 ± 1% [6].

Table 1 reports the initial thickness and the initial mass of coupons. Before immersion, surfaces were rectified by grinding up to SiC emery paper grade 2400. This step had two objectives: (i) to remove the contaminant mechanically after the heat treatment, (ii) to adjust the sample thickness to approximately 1 mm. This thickness was chosen from preliminary tests to consider unidirectional diffusion and planar capacitor approximation later. The sample thicknesses were measured with a micrometric caliper (AsOne, Tokyo, Japan) on 5 positions located at the center and four points of each quarter at the border on the sample surface.

### 2.3. Immersion Tests

The samples were immersed in individual glass containers filled with about 40 mL of a solution composed by 0.1 M NaCl and having a pH of 5.5. Immersion tests were carried out at 35 °C in a controlled temperature chamber. The containers were sealed to avoid the electrolyte evaporation. Gravimetric and permittivity measurements were performed simultaneously along a period of 3 months. Before the analysis, the sample surface was dried with absorbent paper until no water droplets were detected visually. This procedure may slightly underestimate the water quantity however it reduced drastically the gravimetric measurement errors, and avoided the electrical shortcuts during electrical impedance measurements. After experiments, the sample was reinstalled in its container, and the solution’s level was adjusted if necessary, with pure water to keep a constant concentration of NaCl. Two samples were tested per composition, error bars inform about samples variabilities and also take into account measurements imprecisions.

### 2.4. Gravimetric Measurements

Gravimetric measurements were performed with a weight scale (Sefi ITX220, AsOne, Japan) with a precision of 0.1 mg. The relative mass variation given in Equation (7) expresses the water uptake. In first approximation it was assumed that the mass variation came only from the polymer’s water absorption and not to the polymer’s physical degradation. This hypothesis was confirmed since no thickness variation was detected for all samples.

(7) $$\chi_{m}=100\frac{\Delta m}{m_{0}}=100\frac{m_{t}-m_{0}}{m_{0}},$$

where $\chi_{m}$ is the mass percentage of water uptake, $m_{0}$ is the initial mass of the coupon before immersion (reported in Table 1), $m_{t}$ is the mass of the sample after an immersion time t.

### 2.5. Electrical Impedance Measurements for Capacitance and Permittivity Determinations

**Figure 1:**
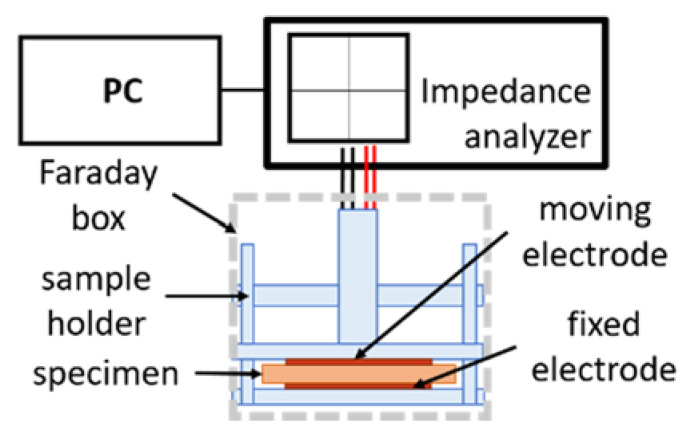
Experimental setup of impedance measurement.

Electrical impedance measurements were performed according to the system in Figure 1. The disc samples were installed on the Controlled Environment Sample holder (CESH) provided by BioLogic and connected to an Impedance analyzer (HP 4194A, Puteaux, France) [16]. In the CESH cell, the gold-plated sensors consisted of two circular electrodes (5 cm^2^) inside a Faraday shield. In such tests, contact quality between the electrodes and the samples is essential to limit artifacts on the measurements [38] The applied force was controlled by the distance between the two electrodes and was kept constant for all the tests performed on each sample. With this setup design, the sample’s aspect ratio (diameter over the thickness) is large enough to neglect the edge effects and polymer deformation is assumed negligible.

Electrical impedance measurements were performed at 0.5 V sweep from 1 kHz to 1 MHz with 11 points per decade. Two samples with same composition were tested to access reproducibility. For data treatments, the relative permittivity of water ($\varepsilon_{water}$) is fixed to 80 for brasher Kingsbury model [27] while the relative permittivity ($\varepsilon_{polymer}$) of the conventional epoxy amine or IL polymers is given about 3 ± 0.5 [39,40]. When the missing rule is applied, the relative permittivity of water is equal to 12 at high frequency and 80 at low frequency using Cole–Cole model [33,34].

### 2.6. Additional Tests

In order to evaluate the polishing effect on the water penetration initiation, surface energies were determined from sessile drop method using Digidrop Contact Angle Meter from GBX Scientific Instruments (Dublin, Irland). Tested liquids are water and diiodomethane. The polar and dispersive components are calculated by using Owens Wendt theory [41,42,43]. Droplets have been measured on both raw surfaces (just after the curing) and polished surface with SiC up to grade 2400.

Mechanical properties have also been investigated by dynamic mechanical analysis (DMA) during the immersion process. Ross et al. have showed that the glass transition temperature (T_g_ in DSC or T_α_ in DMA) is an indicator of water uptake behavior [22]. For the present systems, DMA have been performed at 1 Hz on temperature range from −90 °C to 200 °C at a speed of 3 °C/min. Dimension of rectangular shaped samples are 25 × 5 × 1 mm^3^, and the effective length between the grips is fixed at 10 mm. Maximum applied force is set to 10 N and maximal strain is set to 10 μm.

## 3. Results and Discussion

### 3.1. Contact Angle and Surface Energy

The results of contact angle for water and diiodomethane, total energy with dispersive and polar components are reported in the Table 2. The effect of polishing process on surface energy has been investigated. In the case of epoxy–amine, the polished surface showed a lower surface energy than the raw surface, which means better hydrophobicity. One hypothesis is that the oxide layer formed during the post curing has higher surface energy. Oxygen atoms promote better interaction with water molecules. In one hand, the dispersive component slightly increases from 27.3 mJ/m^2^ to 29.1 mJ/m^2^ after polishing process. In the other hand the polar component drastically decreases from 8.7 mJ/m^2^ to 4.5 mJ/m^2^ after polishing process. In the case of epoxy–IL, polishing process has a higher effect on surface energy, increasing from 18.8 mJ/m^2^ to 31.6 mJ/m^2^. This change is mainly due to the increase in the dispersive component from 18 mJ/m^2^i to28.6 mJ/m^2^. Regarding the polar component is increasing to a lower extand from 0.8 mJ/m^2^ to 3.0 mJ/m^2^.

After the polishing process, both epoxy–amine and epoxy–IL present similar surface energy, with close water contact angles of 84° and 89° for epoxy–amine and epoxy–IL respectively. It means the initiation of water diffusion is similar and water diffusion properties are more linked with the bulk properties. It is worth notice that in this study, samples were polished on purpose and in practical coating applications, non-polished epoxy–IL surface is expected to have much higher hydrophobic properties. Higher hydrophobicity of epoxy–IL will reduce the kinetic of water diffusion initiation, making the latter a good candidate for anti-corrosion coatings.

### 3.2. Dynamic Mechanical Analysis

**Figure 2:**
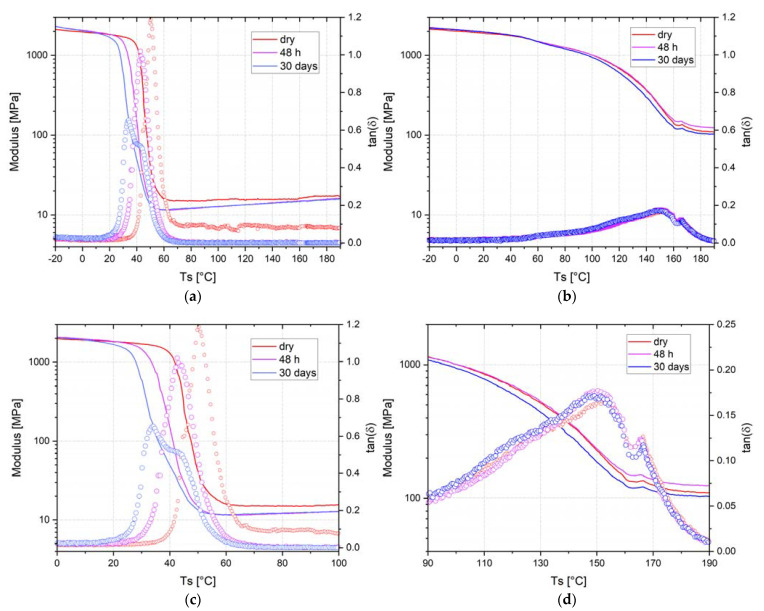
DMA thermogram from −20 °C to 200 °C of (**a**) epoxy–amine and (**b**) epoxy–IL. Zoom at 100 °C span around the T_α_ for (**c**) epoxy–amine and (**d**) epoxy–IL.

According to the literature, these two epoxy networks have the same mechanical performances determined by tensile tests, i.e., Young’s modulus included between 2.6–2.8 GPa and a similar elongation at break of 4.4% [5,44]. In dry condition, T_α_ values for epoxy–IL samples is 150 °C whereas epoxy–amine system is 50 °C. This T_α_ suggests an improving of barrier properties for epoxy–IL. Results for epoxy–amine in Figure 2a,c show after 48 h of immersion, the T_α_ drops to 42 °C After 1 month of immersion, two peaks are observed, a first one at 32 °C and a second at 42 °C. Figure 2b,d are the DMA results of epoxy–IL sample. No evolution of the T_α_ is observable and still the same at 150 °C during the immersion time. For the storage modulus, the values are very close between the epoxy–amine and the epoxy–IL, at 1900 MPa. It is worth noticing that the storage modulus is not affected by water uptake below the T_α_ range of temperature regardless of the immersion time. By contrast, above the T_α_ range of temperature there is a difference of one order of magnitude between epoxy–amine and epoxy–IL. The epoxy–amine system has a storage modulus in the range of 10 MPa whereas epoxy–IL presents a storage modulus around 100 MPa. For these reasons, crosslink density of the resulting epoxy networks denoted νe was also evaluated by using the elasticity theory (Equation (8)) where *R* is ideal gas constant (*R* = 8314 J·K^−1^.mol^−1^), *T_R_* = T_α_ + 30 K, and $E_{r}$ corresponds to the storage modulus in the rubbery state [6,7]. Thus, cross-linking densities of 10,350 mol·m^3^ and 2350 mol·m^3^ were determined for the epoxy–amine and the epoxy–IL networks, respectively. These results can be explained by the higher T_α_ of the epoxy–IL compared to epoxy–D400 systems (150 °C versus 50 °C). This difference in cross-linking density can also explain the better water barrier properties of the ionic liquid epoxy network, but not only, because the chemistry of the networks is totally different: for the epoxy–amine network, a stoichiometric ratio (r = 1) is used and for epoxy–IL network, the ionic liquid is used as an initiator of the polymerization.

(8) $$\upsilon_{e}=\frac{E_{r}}{3\cdot R\cdot T_{r}}$$

### 3.3. Gravimetric Measurements

**Figure 3:**
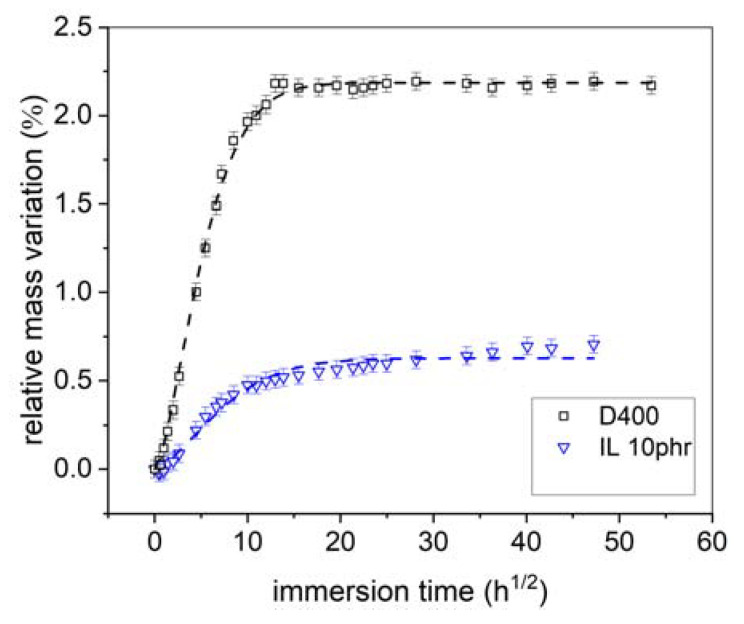
Gravimetric experimental results (symbol) and fitted model using Fick’s Equation (dash lines).

After adsorption, water molecules diffuse in the polymer matrix because of a chemical gradient establishment between the surface and volume. Consequently, water uptake leads to a mass increase depending on polymer molecules interactions with water. Figure 3 reports the relative mass variations with the immersion time in the NaCl solution at 35 °C (Equation (7)). After 30 h^1/2^, a plateau of mass variation is reached whatever the sample, demonstrating that the immersion time was long enough to reach the saturation. The epoxy–amine presents the highest saturation rate at about 2.2 wt.% while for epoxy–IL it is at about 0.6 wt.%. For the diamine, 6 days are needed to charge the polymer with water molecules. It takes only 3 days to reach saturation for epoxy–IL. Therefore, the curing agent plays a significant role on the saturation kinetic and the water uptake depends on the hydrophobicity, wettability. Note that because surfaces were polished for experimental reason, it is not possible to discuss the polymer chemistry effect on the initiation period of water absorption. These results highlight best performances for epoxy–IL with a chemical composition significantly limiting the water uptake. 

To go further with the water uptake quantification, curves were fitted with Equation (2). In Figure 3, an excellent agreement between the experimental and numerical values is observed whatever the samples, which allow to consider the diffusion coefficient of water as a relevant descriptor. The theoretical values of water saturation $(\chi_{\mathrm{sat}}$) and diffusion coefficient (*D_w_*) are reported in Table 3. Quantifications confirm that the epoxy–IL sample is less sensitive to water uptake than epoxy–amine as demonstrated by the lower $\chi_{\mathrm{sat}}$. The use of ionic liquid leads also to a lower coefficient in epoxy–IL sample (3 × 10^−9^ cm^2^·s^−1^) than diamine (9 × 10^−9^ cm^2^·s^−1^). Note that Linde et al. found the same order of magnitude for the water coefficient of diffusion in epoxy diamine sample [45].

### 3.4. Electrical Impedance Analysis Methodology

**Figure 4:**
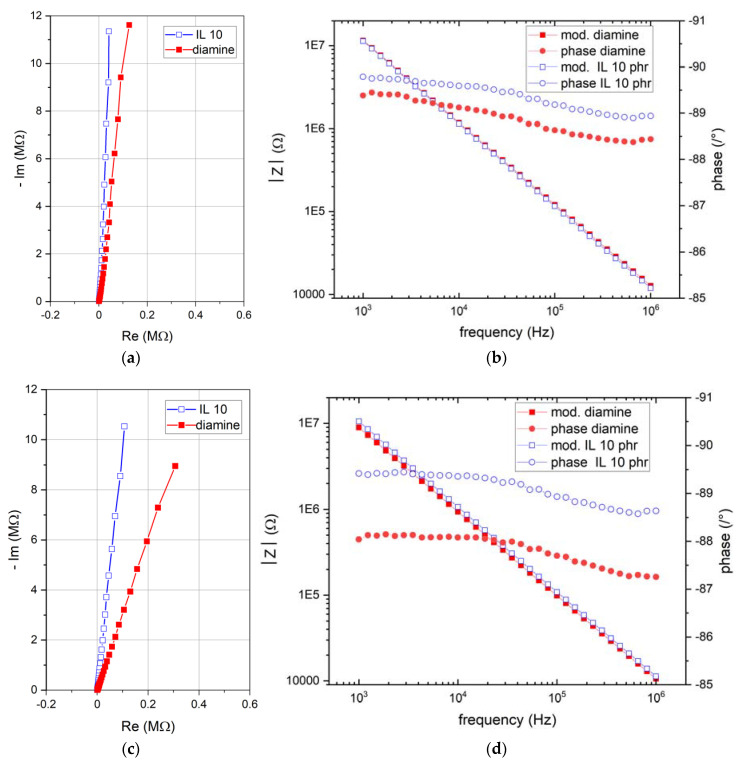
Electrical impedance measurements before immersion (**a**) Nyquist diagram, (**b**) bode diagrams and after 21 days of immersions, (**c**) Nyquist diagrams, and (**d**) bode diagrams.

Electrical impedance measurements were carried out at the same time as the mass measurements. Nyquist diagrams recorded on dry and immersed samples are presented in Figure 4. All curves present a quasi-pure capacitive behavior since the real part of the impedance is negligible compared to the imaginary part. After 7 days of immersion, a drop of the impedance module is observed for all specimens, going from 11.6 MΩ to 9.6 MΩ at 1 kHz for conventional epoxy–amine and 11.4 MΩ to 10.8 MΩ at the same frequency for epoxy–IL 10 phr. The phase angle remains stable around 89°whatever the sample and the immersion time (Figure 3b,d for epoxy–amine and epoxy–IL, respectively).

Capacitance determinations are performed from Nyquist diagrams. Because of quasi pure capacitive behavior, Equation (9) is used to extract the polymer capacitance from parallel capacitor and resistor model [46]. In this work, the capacitance is calculated at 10 kHz which is commonly used for polymer water uptake evaluation [47,48] Then, water uptake from B-K assumptions are calculated from relative permittivity *ε*_10khz_ at one frequency with Equation (3), and using the capacitance values calculated from Equation (9).

(9) $$\varepsilon_{10\mathrm{kHz}}=-\frac{X_{10\mathrm{kHz}}}{C_{0}\omega \left[R_{10\mathrm{kHz}}{}^{2}+X_{10\mathrm{kHz}}{}^{2}\right]}\;at\;f=10\;\mathrm{kHz},$$

where $R_{10\mathrm{kHz}}$ and $X_{10\mathrm{kHz}}$ are the real part and the imaginary parts of the electric impedance, respectively.

Moreover, the water uptake can be determined from the measured relative permittivity using a mixing rule on the whole impedance spectrum. The mixing rule reported in Equation (10) assumes a uniform distribution of permittivity (i.e., uniform diffusion of water) along the sample thickness with two boundaries conditions: the dry polymer permittivity and the water permittivity.

(10) $$\chi \left(t\right)=100\frac{\varepsilon \left(t\right)-\varepsilon_{ini}}{\varepsilon_{wat}-\varepsilon_{ini}},$$

where χ(t) is the volume water uptake in percent, ε(t) is the measured permittivity during the immersion test. $\varepsilon_{ini}$ and $\varepsilon_{wat}$ are the permittivity of the initial condition (dry sample) and water respectively. Note that the permittivity is given at one given frequency.

The material dielectric properties $\varepsilon \left(t\right),\;\varepsilon_{ini}$ are calculated from the complex electric impedance composed by a real part (R) and an imaginary part (X) with Equation (11). Here, ω is the angular frequency (2πf) and $C_{0}$ is the void capacitance. Note that $C_{0}$ depends on the electrode area *S*, the distance between electrodes l, and the free space permittivity ($\varepsilon_{0}$) as shown in Equation (12).

(11) $$Z{\left(t\right)}^{*}=R\left(t\right)+jX\left(t\right)=\frac{1}{j\omega \varepsilon {\left(t\right)}^{*}C_{0}},$$

(12) $$C_{0}=\varepsilon_{0}S/l,$$

In Equation (11), $\varepsilon {\left(t\right)}^{*}$ is the complex relative permittivity defined by a real part $\varepsilon {\left(t\right)}^{\prime }$ and the imaginary part $\varepsilon \;{\left(t\right)}^{\prime \prime }$ as in Equation (13). $\varepsilon {\left(t\right)}^{\prime }$ stands for the electric energy storage, which is a conservative energy, whereas $\varepsilon {\left(t\right)}^{\prime \prime }$ represents the dissipative losses due to electric charge transport. Combining Equations (11) and (13) allows to express the real part and the imaginary part of the relative permittivity as a function of the real part and imaginary part of the electric impedance as in Equation (14). Finally, the water uptake is deduce from Equation (10).

(13) $$\varepsilon {\left(t\right)}^{*}=\varepsilon {\left(t\right)}^{\prime }-j\varepsilon {\left(t\right)}^{\prime \prime },$$

(14) $$\varepsilon {\left(t\right)}^{\prime }=-\frac{X\left(t\right)}{C_{0}\omega \left[R{\left(t\right)}^{2}+X{\left(t\right)}^{2}\right]}\;\mathrm{and}\;\varepsilon {\left(t\right)}^{\prime \prime }=\frac{R\left(t\right)}{C_{0}\omega \left[R{\left(t\right)}^{2}+X{\left(t\right)}^{2}\right]},$$

Figure 4 showed an effect of the water uptake only on the imaginary part of the Nyquist diagrams for the two materials. Therefore, it is interesting to discuss the variations of *ε*(*t*)′ as a function of the immersion time and frequency. Figure 4 displays the real part of relative permittivity of each sample for dry condition and after 20 h and 192 h of immersion. These diagrams show a frequency dependence of the relative permittivity for all specimens, changing with the immersion time and the material chemistry. The IL 10 phr sample presents the lowest frequency dependence in dry conditions with an amplitude variation of about 0.17 between 1 kHz and 1 MHz. In contrast, the amplitude is about 0.42 for the diamine material. After 21 days of immersion, the real permittivity variation between the high frequencies and low frequencies increases. An amplitude of 0.68 is found for the epoxy–amine sample whereas it is about 0.26 for IL 10 phr. It is worth noticing that the permittivity dependence with immersion time becomes more significant at low frequency. It could arise from the mass transport contribution (water diffusion in polymer defects) ort the water permittivity dependance according to the Cole–Cole model.

**Figure 5:**
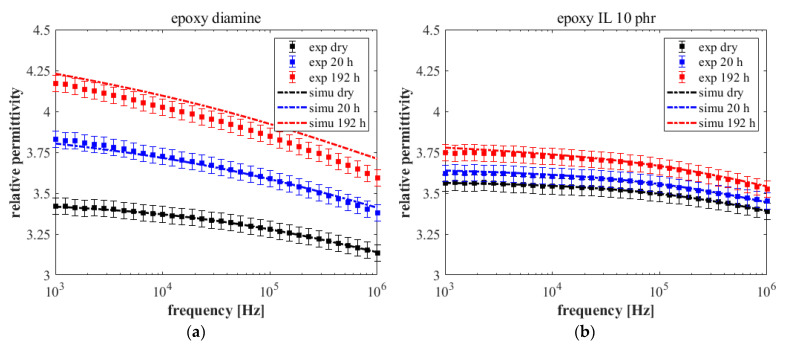
Spectroscopy of the real part of relative permittivity between 1 kHz and 1 MHz, and for 3 different immersion time and simulation results: (**a**) epoxy–amine, (**b**) epoxy–IL.

Experimental data in Figure 5 were fitted using the mixing rules given in Equations (4) to (6). Figure 5 compares the experimental data of dielectric measurement with the numerical modeling. For the epoxy–amine, the model gives a good correlation with the experimental permittivity even though a slight overestimation can be observed for the plot at 192 h (Figure 4a). Similar conclusion is done for epoxy–IL (Figure 4b). Table 4 reports the fitted parameters of the Cole–Cole equation (Equation (4)), equivalent mixing rule (Equation (5)) and water saturation from Equation (6). Note that $\chi_{vol}$ were determined from gravimetric measurements (Equation (6)). 

**Figure 6:**
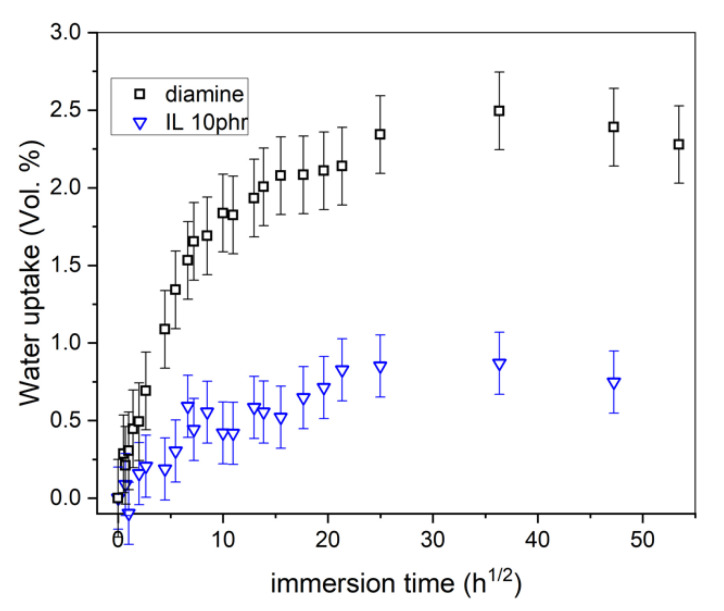
Water uptake in vol.% as a function of the immersion time in NaCl solution for epoxy–amine and epoxy–IL samples using permittivity.

Figure 6 reports the evolution of the water uptake in the two polymers when determined from Equation (10) dealing with permittivity calculation. As from gravimetric measurements, the volumetric percentage of water increases rapidly at the first time of immersion before reaching a limit that is significantly higher in the case of epoxy–amine.

### 3.5. Gravimetric, B-K, and Mixing Rules Comparisons

**Figure 7:**
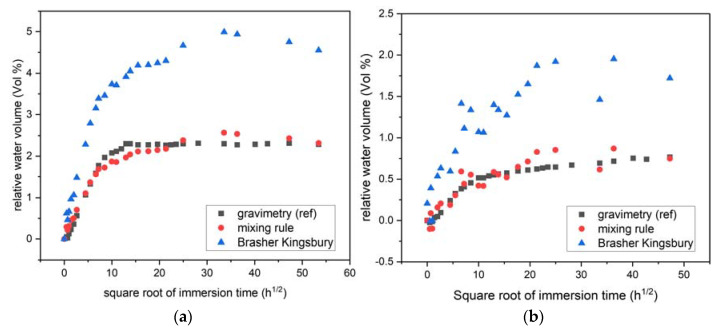
Water uptake as a function of time for (**a**) epoxy–amine, (**b**) epoxy–IL, results comparison between gravimetric, mixing rules, and B-K methods. B-K and mixing rule at 10 kHz.

From gravimetric and impedance measurements, the water uptake was monitored with the immersion time for the epoxy–amine and the epoxy–IL sample. The Brasher and Kingsbury model was applied to calculate the relative volume of water in samples from ε_10kHz_. A conversion of the mass water uptake to the volume water uptake was knowing the volumetric mass of epoxy–amine at (1123 kg/m^3^), which is close from those in the literature [16,49] and epoxy–IL (1134 kg/m^3^):

A comparison of water uptake determined with the three methods is given in Figure 7. Figure 7a reports the relative water volume evolution with the square root of immersion time for epoxy–amine. All the curves follow the same trend with a rapid increase in the water uptake at the initial time of immersion. After 10 h^1/2^ (i.e., 100 h), the water uptake slowdowns and reach a quasi-plateau at 5% with the B-K approximation and 2.5 for the gravimetric measurements and mixing rule analysis. Figure 7b deals with results on epoxy–IL samples. As for epoxy–amine, the water uptake increases significantly at the initial time and starts to stabilize after 10 h^1/2^. A good correlation is observed between the gravimetric results analysis and the permittivity ones when the latter are analyzed with the mixing rule. Since mixing rule data agree with gravimetric measurements, they validate the primary assumption on a two-phase material (polymer–water). One could notice higher fluctuations for B-K and mixing rule results than gravimetric ones. The first hypothesis is a non-perfect parallelism of the surfaces affecting the electrical impedance measurements, whereas gravimetric tests are not sensitive to this parameter. The second hypothesis could be related to different interaction mechanism between the diffused water and residual ionic liquid cluster. Note the latter hypothesis requires further microstructural investigations. 

The B-K approach overestimates the water uptake compared to gravimetric and mixing rule analyses. Such trend has already been reported in Vosgien et al. [21]. For some authors, the B-K equation does not take into account the material swelling, as for the mixing rule applied here. The micrometric caliper measurements reveled a limited swelling characterized by a material thickness increase of 12 µm ± 5 µm and 8 ± 4 µm for the diamine and epoxy–IL samples after 400 h of immersion, respectively. The major different difference between the B-K model and the mixing rule stays in the water permittivity hypotheses. For the Brasher–Kingsbury model, ε_water_ is equal to 80 whatever the frequency, whereas for the mixing rule one should assume a variation of the water permittivity with the frequency which seems to be more realistic [16,50,51,52].

## 4. Conclusions

Gravimetric and electric impedance measurements were applied on immersed polymer materials to evaluate the influence of the curing agents. The water uptake of a conventional epoxy–amine network was compared to one epoxy initiated by one phosphonium ionic liquid (10 phr) after immersion time up to 2500 h (about 3 months). The results can be summarized as:

- Electrical impedance measurements can be successfully applied to detect and to quantify the relative permittivity of polymer materials with the presented mixing rule being applied instead of the Brasher–Kingsbury model, which tends to overestimate the permittivity, thus water uptake.
- Gravimetric and impedance measurements with the novel mixing rule report the same water uptake behavior: both mass and the permittivity increase as the water diffuses in the polymer matrix;
- Ionic liquid used as alternative to amine hardener reduces the water uptake compared to conventional epoxy–amine system: lower diffusion coefficient of water and a lower saturation limit due to better hydrophobic properties but also due to the different chemistry of the networks.

These promising results open new perspectives in the development of anti-corrosion surface coatings. Nevertheless, other epoxy–amine systems with higher T_g_ could be compared to these ionic liquid-based systems. 

## Figures and Tables

**Table 1:** Specimen dimensions, average thicknesses, and initial masses.

Sample	Hardener Ratio (phr)	Average Initial Thickness (mm)	Initial Mass (mg)
epoxy amine	63	1.12 ± 0.03	839.4 ± 0.2
epoxy IL	10	1.14 ± 0.03	905.3 ± 0.2

**Table 2:** Contact angle and surface energy of epoxy amine and epoxy ionic liquid before and after polishing process.

Sample	H_2_O Contact Angle (/°)	CH_2_I_2_ Contact Angle (/°)	Dispersive Part (mJ·m^−2^)	Polar Part (mj·m^−2^)	Total Energy (mj·m^−2^)
Epoxy amine raw	76	53	27.3	8.7	36
Epoxy amine polished	84	53	29.1	4.5	33.5
Epoxy IL raw	106	78	18.0	0.8	18.8
Epoxy IL polished	89	55	28.6	3.0	31.6

**Table 3:** Diffusion coefficient and saturation values for epoxy amine D230 and epoxy IL CYPHOS.

Sample	D_w_ (cm^2^·s^−1^)	$\chi_{sat}$ (wt.%)
epoxy–D400	8.0 × 10^−9^ ± 1	2.2 ± 0.1
epoxy–IL	3.6 × 10^−9^ ± 1	0.6 ± 0.2

**Table 4:** Parameters of mixing rule model.$\;\varepsilon_{0}$ and $\varepsilon_{\infty }$ are the permittivity at low frequency and infinite frequency respectively. $\omega_{c}$ is the cut off frequency and α is the slope coefficient in the Cole–Cole equation. ρ is the density of the specimen and $\chi_{\mathrm{vol}}$ is the maximal water saturation in volume percentage.

Parameters	Epoxy–Amine	Epoxy–IL	Water
$\varepsilon_{0}$	3.45	3.57	80
$\varepsilon_{\infty }$	2.79	3.14	12
$\omega_{c}$ (rad·s^−1^)	2.5π10^6^	3.6π10^6^	200π
α	0.63	0.53	0.87
*ρ* (kg·m^−3^)	1123	1134	1040
$\chi_{vol}$$\;({\%}_{\mathrm{vol}}$)	2.16	0.65	---

## Data Availability

No new data were created or analyzed in this study. Data sharing is not applicable to this article.

## References

[1] May C. (2018). Epoxy Resins: Chemistry and Technology.

[2] Lee H., Neville K. (1967). Handbook of Epoxy Resins.

[3] Ou B., Wang Y., Lu Y. (2021). A review on fundamentals and strategy of epoxy-resin-based anticorrosive coating materials. Polym.-Plast. Technol. Mater..

[4] Cui G., Bi Z., Wang S., Liu J., Xing X., Li Z., Wang B. (2020). A comprehensive review on smart anti-corrosive coatings. Prog. Org. Coat..

[5] Livi S., Silva A., Thimont Y., Nguyen T.K.L., Soares B., Gerard J.-F., Duchet J. (2014). Nanostructured thermosets from ionic liquid building block–epoxy prepolymer mixtures. RSC Adv..

[6] Nguyen T.K.L., Livi S., Pruvost S., Soares B.G., Duchet-Rumeau J. (2014). Ionic liquids as reactive additives for the preparation and modification of epoxy networks. J. Polym. Sci. Part Polym. Chem..

[7] Ionic liquids: A New Route for the Design of Epoxy Networks|ACS Sustainable Chemistry & Engineering. https://pubs.acs.org/doi/abs/10.1021/acssuschemeng.5b00953.

[8] Leclère M., Livi S., Maréchal M., Picard L., Duchet-Rumeau J. (2016). The properties of new epoxy networks swollen with ionic liquids. RSC Adv..

[9] Sonnier R., Dumazert L., Livi S., Nguyen T.K.L., Duchet-Rumeau J., Vahabi H., Laheurte P. (2016). Flame retardancy of phosphorus-containing ionic liquid based epoxy networks. Polym. Degrad. Stab..

[10] Ghali E., Sastri V.S., Elboujdaini M. (2007). Corrosion Prevention and Protection: Practical Solutions.

[11] Talbot D.E.J., Talbot J.D.R. (2001). Corrosion Science and Technology.

[12] Fazende K.F. (2013). Preparation and Detection of Degradation and Chain Scission Events in Epoxy-Amine Networks Using a Profluorescent Nitroxide Probe. Honors Thesis.

[13] Krauklis A.E., Echtermeyer A.T. (2018). Mechanism of Yellowing: Carbonyl Formation during Hygrothermal Aging in a Common Amine Epoxy. Polymers.

[14] Crank J. (1975). The Mathematics of Diffusion.

[15] Gurtin M.E., Yatomi C. (1979). On a Model for Two Phase Diffusion in Composite Materials. J. Compos. Mater..

[16] Ollivier-Lamarque L., Lallart M., Mary N., Uchimoto T., Livi S., Marcelin S., Miki H. (2020). Dielectric analysis of water uptake in polymer coating using spatially defined Fick’s law and mixing rule. Prog. Org. Coat..

[17] Langmuir I. (1918). The adsorption of gases on plane surfaces of glass, mica and platinum. J. Am. Chem. Soc..

[18] Nguyen V.N., Perrin F.X., Vernet J.L. (2005). Water permeability of organic/inorganic hybrid coatings prepared by sol–gel method: A comparison between gravimetric and capacitance measurements and evaluation of non-Fickian sorption models. Corros. Sci..

[19] Shi B. (2020). Connection between dielectric constant and total number of hydrogen-bond groups per cation–anion pair in ionic liquids. J. Mol. Liq..

[20] Ganjaee Sari M., Ramezanzadeh B., Shahbazi M., Pakdel A.S. (2015). Influence of nanoclay particles modification by polyester-amide hyperbranched polymer on the corrosion protective performance of the epoxy nanocomposite. Corros. Sci..

[21] Vosgien Lacombre C., Bouvet G., Trinh D., Mallarino S., Touzain S. (2017). Water uptake in free films and coatings using the Brasher and Kingsbury equation: A possible explanation of the different values obtained by electrochemical Impedance spectroscopy and gravimetry. Electrochim. Acta.

[22] De Parscau du Plessix B., Jacquemin F., Lefébure P., Le Corre S. (2016). Characterization and modeling of the polymerization-dependent moisture absorption behavior of an epoxy-carbon fiber-reinforced composite material. J. Compos. Mater..

[23] Brug G.J., van den Eeden A.L.G., Sluyters-Rehbach M., Sluyters J.H. (1984). The analysis of electrode impedances complicated by the presence of a constant phase element. J. Electroanal. Chem. Interfacial Electrochem..

[24] Hsu C.H., Mansfeld F. (2001). Technical Note: Concerning the Conversion of the Constant Phase Element Parameter Y0 into a Capacitance. Corrosion.

[25] Hirschorn B., Orazem M.E., Tribollet B., Vivier V., Frateur I., Musiani M. (2010). Constant-Phase-Element Behavior Caused by Resistivity Distributions in Films II. Applications. J. Electrochem. Soc..

[26] Amand S., Musiani M., Orazem M.E., Pébère N., Tribollet B., Vivier V. (2013). Constant-phase-element behavior caused by inhomogeneous water uptake in anti-corrosion coatings. Electrochim. Acta.

[27] Brasher D.M., Kingsbury A.H. (1954). Electrical measurements in the study of immersed paint coatings on metal. I. Comparison between capacitance and gravimetric methods of estimating water-uptake. J. Appl. Chem..

[28] Bierwagen G., Tallman D., Li J., He L., Jeffcoate C. (2003). EIS studies of coated metals in accelerated exposure. Prog. Org. Coat..

[29] Sykes J.M. (2004). A variant of the Brasher–Kingsbury equation. Corros. Sci..

[30] Levy O., Stroud D. (1992). Maxwell Garnett theory for mixtures of anisotropic inclusions: Application to conducting polymers. Phys. Rev. B.

[31] Havriliak S., Negami S. (1966). A complex plane analysis of α-dispersions in some polymer systems. J. Polym. Sci. Part. C Polym. Symp..

[32] Havriliak S., Negami S. (1967). A complex plane representation of dielectric and mechanical relaxation processes in some polymers. Polymer.

[33] Cole K.S., Cole R.H. (1941). Dispersion and Absorption in Dielectrics I. Alternating Current Characteristics. J. Chem. Phys..

[34] Cole K.S., Cole R.H. (1942). Dispersion and Absorption in Dielectrics II. Direct Current Characteristics. J. Chem. Phys..

[35] Livi S., Gérard J.-F., Duchet-Rumeau J., Mecerreyes D. (2015). Ionic Liquids as Polymer Additives. Applications of Ionic Liquids in Polymer Science and Technology.

[36] Silva A.A., Livi S., Netto D.B., Soares B.G., Duchet J., Gérard J.-F. (2013). New epoxy systems based on ionic liquid. Polymer.

[37] Soares B., Livi S., Duchet J., Gerard J.-F. (2012). Preparation of epoxy/MCDEA networks modified with ionic liquids. Polymer.

[38] Hao J., Xu X., Taylor N. (2020). Non-contact method to reduce contact problems between sample and electrode in dielectric measurements. High Volt..

[39] Saeedi I.A., Chaudhary S., Andritsch T., Vaughan A.S. (2021). Investigation of the functional network modifier loading on the stoichiometric ratio of epoxy resins and their dielectric properties. J. Mater. Sci..

[40] Yu J., Huo R., Wu C., Wu X., Wang G., Jiang P. (2012). Influence of interface structure on dielectric properties of epoxy/alumina nanocomposites. Macromol. Res..

[41] Owens D.K., Wendt R.C. (1969). Estimation of the surface free energy of polymers. J. Appl. Polym. Sci..

[42] Livi S., Silva A.A., Pereira J., Nguyen T.K.L., Soares B.G., Cardoso M.B., Gérard J.-F., Duchet-Rumeau J. (2014). Supercritical CO_2_–organosilane mixtures for modification of silica: Applications to epoxy prepolymer matrix. Chem. Eng. J..

[43] Van de Grampel R.D., Ming W., van Gennip W.J.H., van der Velden F., Laven J., Niemantsverdriet J.W., van der Linde R. (2005). Thermally cured low surface-tension epoxy films. Polymer.

[44] Yang G., Fu S.-Y., Yang J.-P. (2007). Preparation and mechanical properties of modified epoxy resins with flexible diamines. Polymer.

[45] Linde E., Giron N.H., Celina M.C. (2018). Water diffusion with temperature enabling predictions for sorption and transport behavior in thermoset materials. Polymer.

[46] Hoseinpoor M., Prošek T., Babusiaux L., Mallégol J. (2021). Simplified approach to assess water uptake in protective organic coatings by parallel plate capacitor method. Mater. Today Commun..

[47] Deflorian F., Fedrizzi L., Rossi S., Bonora P.L. (1999). Organic coating capacitance measurement by EIS: Ideal and actual trends. Electrochim. Acta.

[48] Yin X., Hutchins D.A., Chen G., Li W. (2013). Investigations into the measurement sensitivity distribution of coplanar capacitive imaging probes. NDT E Int..

[49] Jang C., Sharifi M., Palmese G.R., Abrams C.F. (2014). Crosslink network rearrangement via reactive encapsulation of solvent in epoxy curing: A combined molecular simulation and experimental study. Polymer.

[50] Kaatze U. (1989). Complex permittivity of water as a function of frequency and temperature. J. Chem. Eng. Data.

[51] Wobschall D. (1977). A Theory of the Complex Dielectric Permittivity of Soil Containing Water: The Semidisperse Model. IEEE Trans. Geosci. Electron..

[52] Schwan H.P., Sheppard R.J., Grant E.H. (1976). Complex permittivity of water at 25 °C. J. Chem. Phys..

